# Biomechanical stress provides a second hit in the establishment of BMP/TGFβ-related vascular disorders

**DOI:** 10.15698/cst2020.02.213

**Published:** 2020-01-20

**Authors:** Christian Hiepen, Jerome Jatzlau, Petra Knaus

**Affiliations:** 1 Freie Universität Berlin, Institute for Chemistry and Biochemistry, 14195 Berlin, Germany.

**Keywords:** BMP, TGFβ, PAH, BMPR2, Integrin, LAP, fibrillin

## Abstract

Cardiovascular disorders are still the leading cause for mortality in the western world and challenge economies with steadily increasing healthcare costs. Understanding the precise molecular pathomechanisms behind and identifying players involved in the early onset of cardiovascular diseases remains crucial for the development of new therapeutic strategies. Taking advantage of CRISPR/Cas9 gene editing in human endothelial cells (ECs), we re-investigated the early molecular steps in a genetic vascular disorder termed pulmonary arterial hypertension (PAH) in our recent study (Hiepen C., Jatzlau J. *et al.*; PLOS Biol, 2019). Here, mutations in the Bone Morphogenetic Protein type II receptor (BMPR2) prime for the hereditary form (HPAH) with downregulated BMPR2 followed by a characteristic change in SMAD signaling, i.e. gain in both SMAD1/5 and SMAD2/3 responses. Remarkably these cells show increased susceptibility to signaling by TGFβ due to remodeling of the extracellular matrix (ECM) and increased biomechanics acting as a secondary stressor for ECs pathobiology. This clearly places BMPR2 not only as a BMP-signaling receptor, but also as a gatekeeper to protect ECs from excess TGFβ signaling.

## BMPR2 MUTATIONS PRIME FOR PAH BUT REQUIRE A SECOND HIT

**Figure 1:**
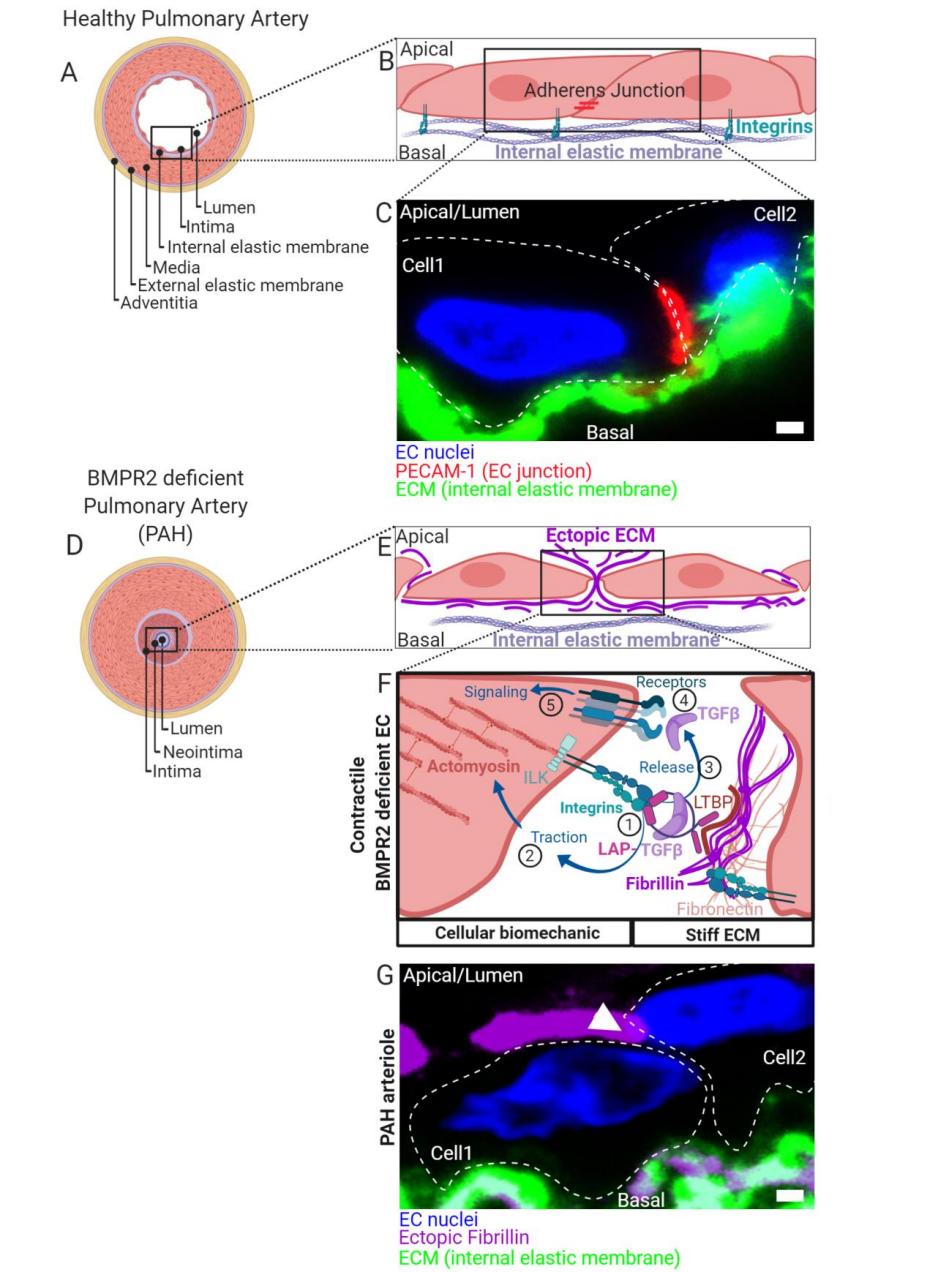
FIGURE 1: **(A)** Healthy pulmonary artery is characterized by a discrete macroscopic organization of tissue and extracellular matrix (ECM) layers (blue) from the inside to the outside of a blood vessel. Facing the lumen, the intima is formed by a thin layer of endothelial cells followed by a discrete ECM layer composing the internal elastic membrane. A big contribution to wall stability is made by the media formed by multiple layers of smooth muscle cells. In small capillaries such as arterioles, the wall thickness is reduced by lesser number of SMC layers. The media is connected to a second discrete layer of ECM composing the external elastic membrane facing an outer layer of cells and ECM referred to as adventitia. **(B)** ECs facing the lumen with their apical side connect to neighboring cells through distinct contact sites and to the internal elastic membranes by integrin-rich focal adhesions. Complexes of integrins are composed of α/β-subunits. Shown are adherens-junctions (red) at contact sites and integrin complexes engaging with ECM proteins of the basal lamina of the internal elastic membrane. **(C)** Immunohistochemical fluorescence staining of transversal cross-sectioned human pulmonary arterioles. The region of interest shown depicts on two adjacent EC nuclei (blue) from cells constituting the intima with junctional marker protein platelet endothelial cell adhesion molecule-1 (PECAM-1) shown in red together with the internal elastic membrane in green. Cell body boundaries as slash dotted lines. **(D)** BMP receptor type II (BMPR2) deficiency primes for pulmonary arterial hypertension (PAH). During PAH, the discrete macroscopic and microscopic organization of the tissue layers lining the blood vessel lumen is disturbed. Histologically most pronounced and dependent on the disease stage, the media thickens by hypertrophy of SMCs. Moreover, internal and external elastic membranes remodel, external cells invade and excessive ECM deposits are found ectopically. The lumen is obstructed and a neointima is formed. At later stages, vascular lesions can form (not shown) where discrete tissue borders disappear. The molecular details and the contribution of intimal thickening and neointima formation are less defined. Trans-differentiation of ECs towards a myofibroblast-like cell type is suggested. **(E)** We found that BMPR2-deficient ECs deposit ectopic ECM in their cell-junctions (fibrillin) and that the general composition of the ECM (Matrisome) changes. **(F)** In the absence of BMPR2, ECs form active mechano-complexes containing β1-integrins and Integrin-linked kinase (ILK) at cell contact sites on the expense of VE-Cadherin and PECAM-1 localization. The ectopic ECM is decorated with the latency associated peptide (LAP) bound TGFβ. (1) In BMPR2-deficient cells β1-integrin-ILK containing mechanocomplexes bind to a RGD-motive within the latency associated peptide (LAP) of TGFβ. Latent TGFβ binding to the ECM is facilitated by increased formation of fibronectin fibers acting as a template for fibrillin fibrils to which the latent-transforming growth factor beta-binding protein (LTBP) facilitates the LAP (TGFβ) tethering. In its ECM-bound form, this latency complex renders TGFβ to be biologically not accessible for cells. (2) Intracellular traction forces are generated by RHO kinase dependent actomyosin contractility. The contractile actomyosin cytoskeleton connects to integrins via the adaptor protein ILK. (3) Mature TGFβ is released from LAP by integrin binding and actomyosin contraction. Conformational changes of the latency complex expose protease cleavage sites allowing for the retrieval of soluble mature TGFβ. ECM stiffness is pivotal to this process providing resistance to the cell-generated traction. (4) Mature TGFβ is able to bind to BMP/TGFβ receptor complexes comprising high affinity TGFβ receptor type II. Activated receptor complexes induce intracellular signalling cascades of which we have studied SMAD-dependent signaling in detail in *Hiepen C, Jatzlau J. et al. PLoS Biol 2019*. The mechanical aspects of this mechanism is suppressed in ECs, when BMPR2 is present, making it an important gatekeeper molecule. **(G)** Immunohistochemical fluorescence staining of transversal cross-sectioned pulmonary arterioles from a PAH donor. The region of interest shown depicts on two adjacent EC nuclei (blue) from the neointima with ectopic fibrillin deposits at the basal and apical side and the internal elastic membrane shown in green. Ectopic fibrillin deposits at cell contact sites, white arrow head. Cell body boundaries, slash dotted lines. Scale bar is 2μm.

While *BMPR2* mutations appear as one of the main genetic drivers for HPAH, low *BMPR2* expression is also found in idiopathic cases (IPAH). Therefore, restoring BMPR2 expression became a wishful thinking in the design of PAH treatment with new attempts in current clinical pipelines. It is proposed that mutant BMPR2 is not sufficient to cause PAH and that an additional stressor is needed for disease onset following the “second-hit” concept known from cancer pathologies. A healthy pulmonary artery is characterized by a discrete tissue architecture with EC-derived intima, an internal elastic membrane (iem) composed of a defined mixture of ECM proteins, followed by multiple layers of smooth muscle cells (SMCs) separating the outer adventitia **(Fig. 1A)**. EC attachment to the iem is facilitated at the basal side by integrin complexes engaging with the ECM, while junctional cell-to-cell contacts are established by intermolecular interaction of e.g. adherence junction proteins (**Fig. 1B, C**). Early pathomechanisms in PAH are media hypertrophy, intima thickening, neointima formation **(Fig. 1D)** and obstruction of small arterioles leading to increased blood pressure in the lung. This will consequently challenge the right heart ventricle. Amongst the secondary stressors proposed are imbalanced hormones, acute inflammation, hypoxia, protein misfolding with ER stress and epigenetic triggers. In search for secondary stressors that are conceptually different to the ones proposed, we speculated that the mechanical interaction of ECs with their extracellular environment could be of similar importance. We proposed that loss of BMPR2 drives cells towards a prestressed biomechanical state, allowing them to initiate a vicious feed-forward TGFβ auto-stimulation cycle.

## BMPR2 DEFICIENCE FAVORS BIOMECHANICAL TGFβ RETRIEVAL FROM THE ECM

We could show, that *BMPR2*-deficient cells change their biomechanics via differential expression and ectopic depo-sition of ECM components such as fibrillin-1 and fibronectin **(Fig. 1E)**. This gives rise to a mechanical micro-milieu permissive to harbor high amounts of the latent and biologically inactive form of TGFβ (latent TGFβ). Latent TGFβ is bound to a latency associated peptide (LAP) and tethered to fibrillin microfibrils **(Fig. 1F)**. First, binding of cell-surface integrins to the RGD sequence within LAP of TGFβ (1) is followed by actomyosin-dependent traction forces (2) which mechanically activate and release mature TGFβ (3). We found that BMPR2-deficient cells express high levels of activated RDG binding β1-integrin and show increased Rho kinase-dependent contractility. By this mechanism, mature and biologically active TGFβ is released from its extracellular depot to thus bind to TGFβ-receptor complexes on the cell surface (4) and to induce cellular signaling in an auto-stimulatory manner (5) **(Fig. 1F)**.

## ENDMT AND JUNCTIONAL REORGANIZATION IN RESPONSE TO BIOMECHANICAL STRESS

Excessive TGFβ signaling in ECs induces trans-differentiation towards a mesenchymal-like phenotype, a process termed endothelial-to-mesenchymal transition (EndMT). Indeed, in BMPR2 deficient cells we found structural alterations such as increased actin stress-fiber formation and changes in stress fiber stiffness and subcellular localization, together with increased expression of mesenchymal markers. Interestingly, in the absence of BMPR2 we could localize both β1-integrin and integrin-linked kinase (ILK) at cell contact sites culminating in major changes in the junctional architecture highlighted by the loss of VE-Cadherin and PECAM-1. In fact, we found resolution of EC junctions accompanied by ectopic fibrillin deposits that atypically span through cell contact sites to project apically **(Fig. 1G)**. When cultured under steady state conditions, BMPR2-deficient cells were mechanically capable for the retrieval of active TGFβ from its latent ECM depot. Ectopic fibrillin deposits and signs of EndMT were also found in the intima regions of human pulmonary arteries from PAH patients **(Fig. 1G)**. Thus, it is possible that the mechanically driven auto-stimulation with TGFβ contributes to EndMT in PAH. Biomechanical driven TGFβ release and ectopic ECM production is a process known from scar formation and fibrosis and is primarily a feature of fibroblasts. Indeed, our findings confirm that EndMT of BMPR2-deficient ECs gives rise to a myo-fibrotic cell type, which is potently auto-stimulated by TGFβ. Mechanical TGFβ release and auto-stimulation seems to promote the disease onset at the EC layer in blood vessels. Thus, biomechanics acts as a bona fide secondary stressor in this process.

## COMMONALITIES AND OUTLOOK ON BMP/TGFβ RELATED VASCULAR DISORDERES

Interestingly, vascular diseases such as hereditary hemorrhagic telangiectasia (HHT), Marfan syndrome and Loeys-Dietz-syndrome may be conceptually related. Even though clinically distinct, PAH, HHT, Marfan- and Loeys-Dietz-syndrome may be driven by a similarly occurring biomechanical stressor because they show dysregulation of the same molecular players found in BMPR2-deficient ECs (i.e. TGFβ, BMP9, endoglin, ALK1, Alk5, fibrillin and SMADs). While the molecules involved appear conserved, the biomechanics of each vascular bed in which clinical symptoms develop is very distinct ranging from aorta to small arterioles and capillaries. It would be therefore very important to identify commonalities between these vascular diseases and to develop translational approaches implementing experts in biophysics, biochemistry and medicine. Further action has to be taken to unveil if our findings have clinical relevance. It raises the question whether a direct targeting of cellular biomechanics i.e. “making cells more relaxed” should be considered when developing new therapeutic intervention strategies for PAH.

## Abbreviations:

BMP: – Bone Morphogenetic Protein;
BMPR2: – BMP Receptor Type II;
EC: – endothelial cell;
ECM: – extracellular matrix;
EndMT: – endothelial-to-mesenchymal transition;
HHT: – hereditary hemorrhagic telangiectasia;
HPAH: – hereditary PAH;
iem: – internal elastic membrane;
IPAH: – idiopathic PAH;
LAP: – latency associated peptide;
PAH: – Pulmonary Arterial Hypertension.

